# Metacognition tracks sensitivity following involuntary shifts of visual attention

**DOI:** 10.3758/s13423-022-02212-y

**Published:** 2022-11-16

**Authors:** Samuel Recht, Pascal Mamassian, Vincent de Gardelle

**Affiliations:** 1 Department of Experimental Psychology, University of Oxford, Anna Watts Building, Oxford, OX2 6GG UK; 2 Laboratoire des systèmes perceptifs, Département d’études cognitives, École normale supérieure, PSL University, CNRS, 75005 Paris, France; 3 CNRS and Paris School of Economics, Paris, France

**Keywords:** Attention, Metacognition, Confidence

## Abstract

Salient, exogenous cues have been shown to induce a temporary boost of perceptual sensitivity in their immediate vicinity. In two experiments involving uninformative exogenous cues presented at various times before a target stimulus, we investigated whether human observers (N = 100) were able to monitor the involuntary increase in performance induced by such transients. We found that an increase of perceptual sensitivity (in a choice task) and encoding precision (in a free-estimation task) occurred approximately 100 ms after cue onset, and was accompanied by an increase in confidence about the perceptual response. These simultaneous changes in sensitivity and confidence resulted in stable metacognition across conditions. These results suggest that metacognition efficiently tracks the effects of a reflexive attentional mechanism known to evade voluntary control, and illustrate a striking ability of high-level cognition to capture fleeting, low-level sensory modulations.

**Supplementary Information:**

The online version contains supplementary material available at 10.3758/s13423-022-02212-y.

## Introduction

Sometimes, the saliency of an event (e.g., a bee hovering too close) or the meaning of a signal (e.g., a finger pointing at a snake) is such that we quickly disengage from the ongoing task to reallocate our attention elsewhere. Selective spatial attention has been defined as the prioritization and enhancement of a stimulus at a particular location (Carrasco, 2011; Nobre & Kastner, 2014; Posner, 1980). Two forms of attention have classically been distinguished. Endogenous attention is goal-directed and prioritizes information that is deemed relevant for the observer. It has a slow deployment rate (~300 ms) but can be sustained in time. By contrast, exogenous attention enables an organism to react quickly and automatically to a potential threat: it is a fast (~100 ms) and involuntary, albeit short-lasting process (Carrasco, 2011; Nobre & Kastner, 2014). In psychophysical experiments, exogenous orienting is triggered by sharp contrast transients in the vicinity of a target (Carrasco, 2011; Solomon & Morgan, 2018), the latter then being reported more quickly (Jonides & Irwin, 1981; Posner, 1980) and more accurately (Carrasco, 2011), even when the cue is uninformative (Ling & Carrasco, 2006; Nakayama & Mackeben, 1989; Remington, Johnston, & Yantis, 1992).

Since spatial attention affects perceptual performance, knowing whether attention was deployed is a good indication of the quality of one’s own perception. This *metacognitive* knowledge is useful to regulate behavior: a driver, for example, may decide to slow down if unsure about the color of a traffic light. The subjective estimation of a decision’s accuracy about a visual stimulus can be probed experimentally using confidence judgments. How well confidence judgments track performance is also known as metacognitive ability, or simply metacognition (Mamassian, 2016). Whether metacognition can monitor the fluctuation of performance linked to attention is, however, still unclear.

While the effect of attention on metacognition has been considered in the literature, the findings are mixed: some studies showed dissociations between accuracy and confidence during manipulation of spatial attention (Kurtz et al., 2017; Wilimzig et al., 2008) or temporal attention (Recht, Mamassian, & de Gardelle, 2019). Other studies suggested that spatial attention increases both sensitivity and confidence (Denison et al., 2018; Zizlsperger et al., 2012). Most of these studies, however, considered endogenous orienting of attention.

Metacognition is usually depicted as a high-level process: merely under voluntary control, it could potentially share some of the neuronal bases involved in the orienting of endogenous attention (Gilbert & Li, 2013). Its relation to exogenous attention, however, is much less clear: it could even be argued that exogenous attention should evade metacognitive monitoring as much as voluntary control. On one hand, because of its unrepressed nature, exogenous attention could impede metacognition by disrupting high-level cognitive monitoring. On the other hand, the change in signal induced by exogenous attention being largely bottom-up and transient, its effects may simply remain unnoticed by metacognition. These predictions are consistent with the results of one study that found no effect of involuntary attention on confidence (Kurtz et al., 2017).

Another contrasting view depicts metacognition as strongly yoked to the sensory evidence used in perception (e.g., Kiani & Shadlen, 2009), implying that the effect of exogenous attention should be reflected in metacognition. A recent study investigating the combined effects of exogenous and endogenous attention found that metacognition adequately reflected changes induced by attention (Landry et al., 2021). However, no study to date has considered the effect of exogenous attention on confidence by contrasting valid/invalid non-predictive cues exclusively. Assessing whether confidence tracks such a fleeting attentional mechanism should provide important insights on the versatility and limits of metacognitive monitoring.

To arbitrate between these two views, we used an exogenous pre-cueing approach combined with confidence judgments. Participants categorized the orientation of a low contrast Gabor patch (Experiment 1; e.g., Pestilli & Carrasco, 2005) or estimated the orientation of a “clock” (Experiment 2) briefly presented at one of two locations, and then indicated their confidence. The target stimulus was preceded by a peripheral pre-cue, unpredictive of the target’s location, and the onset asynchrony between the cue and the target (hereafter CTOA) was varied. We hypothesized that if confidence could track sensitivity, we should observe a positive effect of valid exogenous pre-cues on confidence mostly at short CTOAs. We also investigated how accurately confidence judgments reflect task performance (i.e., metacognition).

For both experiments, we found evidence that confidence efficiently tracks the involuntary, short-lasting gain in sensitivity induced by exogenous attention. Notably, the deployment of exogenous attention did not disrupt metacognition, which remained stable across all experimental conditions. These results suggest that metacognitive monitoring is able to process the effects of certain low-level sensory modulations occurring beyond the realm of voluntary control.

## Experiment 1

### Material and methods

#### Participants

Ten right-handed participants were recruited from the French RISC pool of participants. The sample size was estimated from the validity effect size of two previous studies involving a similar exogenous paradigm. White, Lunau, and Carrasco (2014) found an effect size of *d* = 1.13, therefore requiring N = 10 to achieve 85% power in a two-tailed t-test. Liu, Pestilli, and Carrasco (2005) found η2 = 0.65, requiring N = 8 to achieve 85% power. We therefore chose N = 10 as a target. This choice is also consistent with a more recent study reporting *d* = 1.16 (requiring N = 10) with the same paradigm (Fernández, Li, & Carrasco 2019). Estimates were conducted using G*Power. Participants provided informed written consent prior to the experiment and received 30 euros for their time. The experiment was divided into three sessions of 1 h each, over 3 different days. The experimental procedure received approval from the Paris School of Economics (PSE) ethics review board.

#### Stimuli

Target and distractor consisted in two 2° Gabor patches (spatial frequency: 5 c/°; fixed 12% contrast) with Gaussian envelope. The target was oriented either clockwise or counter-clockwise relative to vertical; its orientation was calibrated beforehand for each participant to reach a 75% average accuracy in the main task (see *Calibration* section below). The distractor was always horizontal. The target and the distractor were displayed at 5° eccentricity from the center of the screen, on the horizontal midline, one on each side of the screen. A 0.4° fixation dot was presented at the center of the screen. The pre-cue consisted of a 2° black line displayed 1.5° above the target/distractor center. Stimuli were presented on a gray background. The experiment was programmed using Python and the PsychoPy toolbox (Peirce, 2007), and ran on a computer running Linux Ubuntu.

#### Procedure

Participants sat in a dark room during the experiment, 57 cm from the screen (CRT monitor, 1,920 × 1,080 pixels, 100-Hz refresh rate), with their head maintained using a chinrest. After a 200-ms inter-stimulus interval (ITI), each trial started with the fixation dot displayed for a duration sampled from an exponential decay (scale: 500 ms, bounded within the [300, 1,000] ms interval). This was done to maximize temporal uncertainty about stimuli onset. At the end of this delay, the pre-cue was presented for 60 ms. After a variable cue-to-target onset asynchrony (five different CTOA conditions: 100, 150, 250, 450, and 850 ms, equally spaced in logarithmic scale), both target and distractor were displayed on either side of the fixation dot for 30 ms. Participants were informed that the target was always the non-horizontal Gabor. Participants were asked to categorize the target as clockwise versus counter-clockwise (Type 1 decision) and press the corresponding key on the keyboard (left arrow for counterclockwise, right arrow for clockwise). In 50% of the trials, the target appeared at the same location as the cue (“valid” condition), and for the remaining trials at the opposite location (“invalid” condition). After their response, participants were prompted to report their confidence in their response using the up/down arrow keys (Type 2 decision): *is your confidence for this trial higher or lower than average?* We reasoned that this form of confidence judgment would encourage participants to report high and low confidence in a balanced manner over the whole experiment, which would be beneficial for our statistical analyses. Participants started with ten practice trials with feedback prior to the calibration (see below), which was then followed by the main experiment. Participants were provided with a 10-s break every 60 trials. The design was fully factorial with 5 CTOAs conditions × 2 pre-cue conditions (valid/invalid), with pseudo randomization per virtual blocks of 20 trials.

Participants were instructed to fixate the center of the screen during the whole trial period, given that target location was unpredictable. The cue was fully unpredictive, and participants had no further incentives to orient their attention voluntarily towards the cued location. As such, no eye-tracking monitoring was used in the present study, but it is reasonable to assume that participants maintained their gaze at the center to maximize their chance to properly discriminate the target. We cannot exclude that a small proportion of the trials might have been affected by incorrect fixation. Although the pattern of results of Experiment 1 suggests that participants did not move their eyes towards the cued location even at longer CTOAs, it might account for some of the negligible evidence observed in the long CTOA condition. Participants completed three sessions of 1 h each, with 560 trials per session (1,680 trials in total).

#### Calibration

The psychometric function relating orientation discrimination (the proportion of “counterclockwise” responses) to target orientation was estimated prior to the beginning of the experiment for each participant in order to aim for a 75% average perceptual accuracy in the main task. From the participant's perspective, the task during this calibration part looked similar to the one in the main experiment, but the orientation of the target was varied from trial to trial using an Accelerated Stochastic Approximation (ASA) staircase procedure (Kesten, 1958). In the calibration part, the cue was systematically displayed on both the target and the distractor side, CTOA was fixed at 100 ms, and confidence judgments were not requested. At the end of the calibration, the psychometric curve was estimated using Maximum Likelihood Estimation (MLE), to extract angle values (separately for clockwise and counterclockwise targets) leading to 75% accuracy. These values were then kept constant for the main task, to reduce the risk of inflating metacognitive ability estimates (Rahnev & Fleming, 2019).

**Fig. 1:**
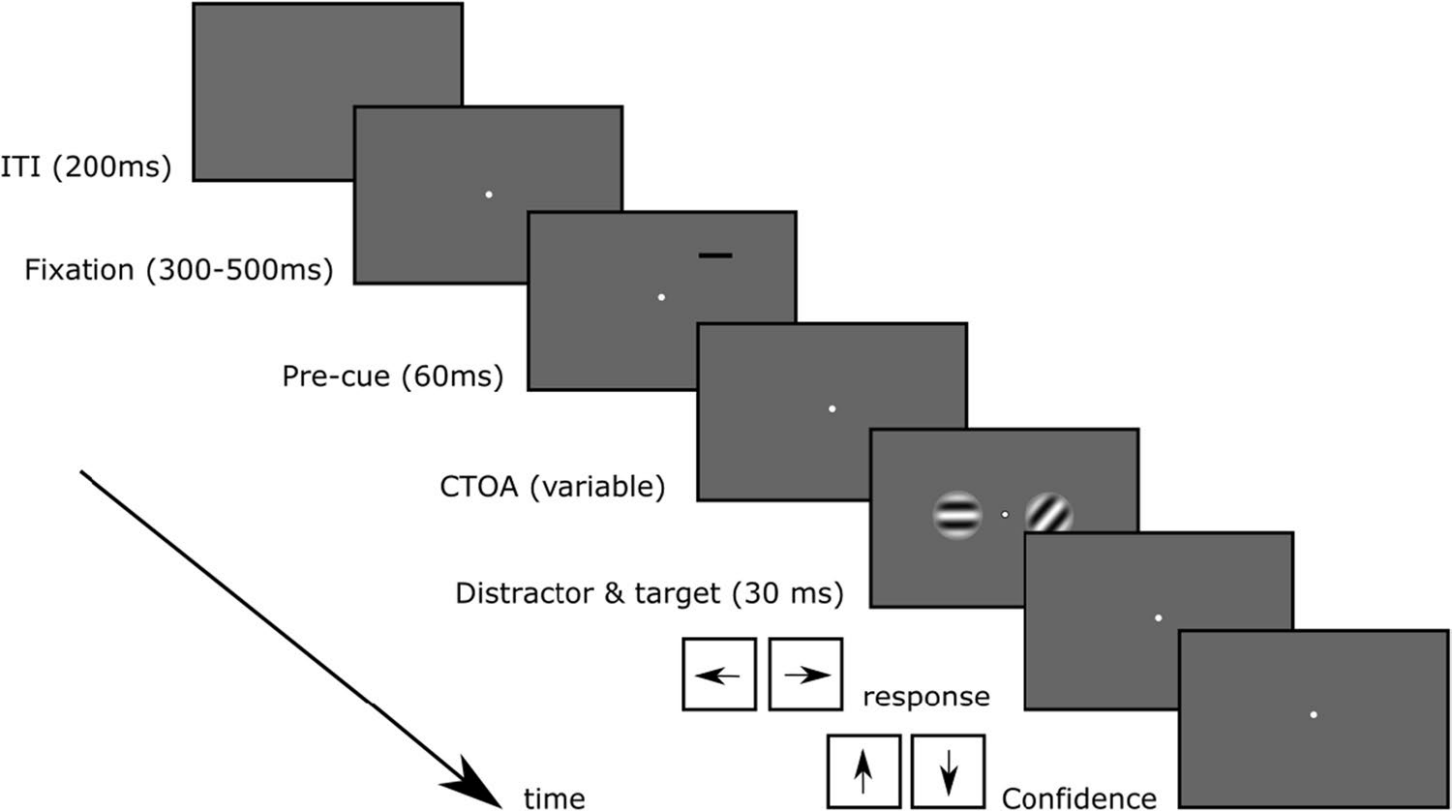
Experimental protocol for Experiment 1: On each trial, after a random delay, a cue is briefly presented on one side of the fixation cross. After a variable cue-to-target onset asynchrony (CTOA), a target and a distractor are presented. The target can appear slightly below the cue (valid condition, as illustrated here) or on the opposite side (invalid condition). The target is an oriented Gabor patch (either clockwise or counter-clockwise) and the distractor is a horizontal Gabor patch. After target and distractor offset, the participant has to discriminate target orientation (clockwise or counter-clockwise) and then rate their confidence in this response on a 2-point scale (more or less confident than average)

Figure 1 shows the experimental protocol for Experiment 1.

#### Measures

We were interested in estimating both perceptual (Type 1) and meta-perceptual (Type 2) sensitivities. We thus used Signal Detection Theory (SDT) to estimate Type 1 sensitivity (d'), which provided us with a bias-free measure of accuracy (Green & Swets, 1966; Macmillan & Creelman, 2005). Trials were grouped using the clockwise-oriented category as signal, leading to four categories of trials: (a) hits, where a CW target was correctly reported as CW; (b) misses, in which a CW target was reported as CCW; (c) false alarms, where a CCW target was reported as CW; (d) correct rejections, where CCW was reported as CCW. This grouping was conducted for each participant and each condition separately, and sensitivity (d') was calculated as the difference in z-scores between the hit rate and the false alarm rate.

As a proxy for Type 2 sensitivity (that is, how well confidence ratings relate to objective accuracy), we used the Meta-d’/d’ ratio, as it is less prone than other measures to shifts in Type 1 sensitivity or response bias. Meta-d’ corresponds to the Type 1 sensitivity that would produce the collected Type 2 (or confidence) responses, if the observers were optimal at the metacognitive level (Maniscalco & Lau, 2012). This value, the meta-d’, can then be compared to the actual sensitivity (d') objectively measured for each participant. In particular, the meta-d' is equal to the d' when the participant has optimal metacognitive access to Type 1 decision information. The ratio meta-d'/d', or “m-ratio” is referred to as metacognitive efficiency. To investigate the effect of cueing on metacognitive efficiency, we thus considered the m-ratio, after estimating d’ and meta-d’ using Maximum Likelihood methods. This procedure was applied for each participant, CTOA and pre-cue validity separately.

#### Analyses

For clarity, and because we were interested in within- not between-participant variability, the error bars in the following figures are based on the 95% confidence interval (CIs) of the within-participant variability. These CIs were calculated using the Cousineau-Morey intervals (Baguley, 2012; Cousineau, 2005; Morey, 2008). All the analyses were carried out using the R programming language (version 4.0.4, R Core Team, 2013). When necessary, ANOVAs were corrected using the Greenhouse-Geisser adjustment and t-tests were corrected using the Welch-Satterthwaite adjustment. We report the V-statistic from Wilcoxon’s signed rank test using uppercase an T when the Shapiro-Wilk normality test failed, and Student’s t-statistic using lowercase a t otherwise. Bayes factors were calculated using the “ttestBF” functions for t-tests, the “correlationBF” for correlation test, and the “anovaBF” function for ANOVAs, from the BayesFactor R package (version 0.9.12-4.3, Morey & Rouder, 2018). The Bayes factor for interactions in ANOVAs was estimated by comparing a model with the main effects to a model with both the main effects and the interaction. For the Wilcoxon signed rank tests, the Bayes factors were estimated using JASP (version 0.16.1.0). For all analyses, we used the default prior distribution provided with the package. We always report the Bayes factor in favor of the alternative hypothesis (BF_10_), with values above 3 providing evidence in favor of the alternative hypothesis and values below 0.33 evidence in favor of the null. Meta-d’ values were estimated using the “fit_meta_d_MLE” function from the “metaSDT” R package (version 0.6.0).

### Results

#### Exogenous pre-cues affect performance and confidence at short CTOA

We first evaluated how performance and confidence were affected by exogenous pre-cues, with separate ANOVAs for sensitivity, response times (RTs), and average confidence as successive dependent variables, and with pre-cue validity and cue-to-target onset asynchrony (CTOA) as independent variables.

**Fig. 2:**
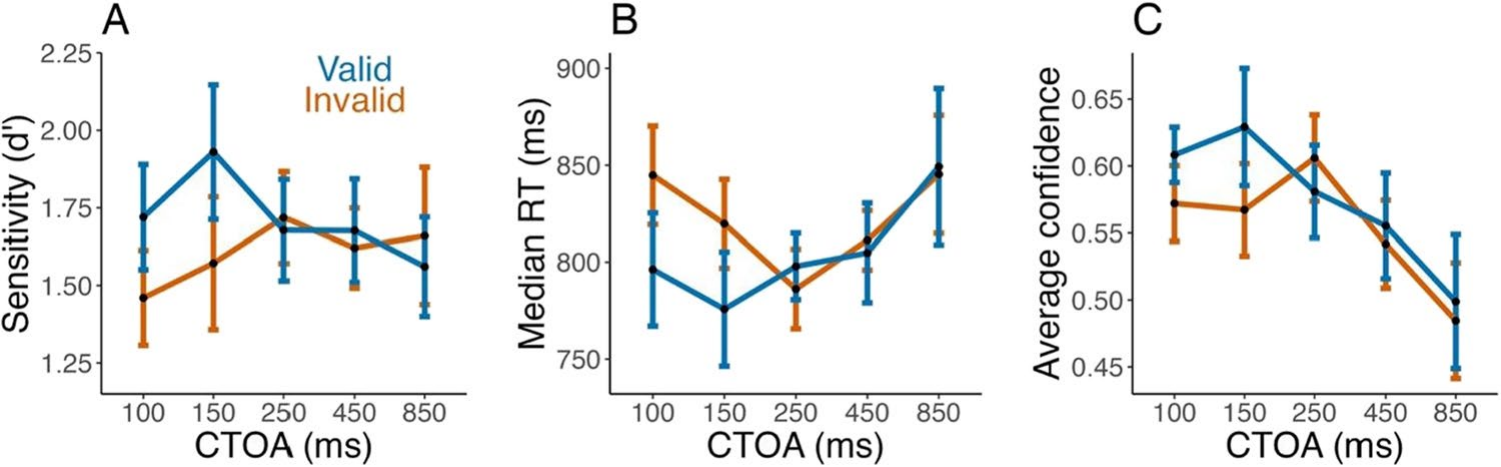
Cueing effects (Experiment 1): (**A**) Average sensitivity (d’) as a function of cue-to-target onset asynchrony (CTOA, equally spaced in logarithmic scale) for valid (blue) and invalid (orange) cues. Sensitivity is greater at valid locations for short CTOA. (**B**) Median response time as a function of CTOA and cue validity, with lower response times for valid location, short CTOA. (**C**) Average confidence as a function of CTOA and cue validity. The 100-ms CTOA showed greater confidence for valid cues. Error bars represent within-subjects 95% confidence intervals

For sensitivity, we found a significant interaction between CTOA and validity (*F*(3.2,28.8) = 4.25, *p* = 0.012, *g* = 0.08, BF_10_ = 0.89), with no significant main effect of CTOA (*F*(2.9,26.3) = 1.18, *g* = 0.04, *p* = 0.334, BF_10_ = 0.12) or validity (*F*(1,9) = 3.7, *g* = 0.03, *p* = 0.088, BF_10_ = 0.76). Paired Wilcoxon tests at each CTOA confirmed a significant gain in sensitivity for the valid compared to the invalid condition at short CTOAs (100 ms: *T*(9) = 52, *p* = 0.0098, *r* = 0.79, BF_10_ = 21.48; 150 ms: *T*(9) = 50, *p* = 0.020, *r* = 0.72, BF_10_ = 7.52) and some evidence for an absence of effect at longer CTOAs (all *p* > 0.30, BF_10_ < 0.36). These results confirmed that our cueing procedure successfully triggered exogenous attention (Fig. 2A).

To discard a potential speed-accuracy trade-off, we examined median response times (Fig. 2B), which exhibited the same pattern as sensitivity did. The repeated-measures ANOVA showed an effect of CTOA (*F*(2.0, 18.2) = 5.41, *g* = 0.01, *p* = 0.01, BF_10_ = 0.06), no effect of validity (*F*(1,9) = 3.4, *p* = 0.1, *g* = 0.003, BF_10_ = 0.23), but an interaction (*F*(2.54, 22.87) = 5.10, *p* = 0.01, *g* = 0.01, BF_10_ = 0.09). However, Bayes factors were strongly favoring the null for both CTOA and the interaction. These results demonstrate that the effect of exogenous attention on sensitivity was not the result of a speed-accuracy tradeoff.

Confidence was affected similarly (Fig. 2C). The ANOVA showed a main effect of CTOA (*F*(2.1,18.6) = 10.11, *g* = 0.09, *p* = 0.001, BF_10_ = 0.87), no effect of validity (*F*(1,9) = 3.9, *g* = 0.006, *p* = 0.079, , BF_10_ = 0.27), but an interaction between CTOA and validity, despite the Bayes factor providing moderate evidence in favor of the null (*F*(2,18.1) = 4.07, *g* = 0.01, *p* = 0.034, BF_10_ = 0.12). Paired Wilcoxon tests at each CTOA confirmed a higher confidence for the valid than for the invalid condition at 100 ms CTOA (*T*(9) = 48, *d* = 0.66, *r* = 0.037, BF_10_ = 3.45, Wilcoxon test), but not for other CTOAs (p > 0.08). In other words, confidence and performance both increased for valid trials at short CTOAs. In addition, we note confidence decreases with CTOAs, which might reflect the increase in response times at longer CTOAs (this effect is unlikely due to temporal expectations, given the higher proportion of short CTOAs). Of note, while sensitivity was boosted for both 100 ms and 150 ms CTOAs, confidence was only significantly boosted at 100 ms. This discrepancy might potentially be due to a lack of power, given that second-order ratings like confidence are usually noisier than first-order responses (Mamassian, 2016).

To confirm the similarity between sensitivity and confidence, we calculated the cueing effect (valid minus invalid) for confidence and sensitivity at each CTOA, and evaluated Pearson’s correlation across CTOAs for each participant. As expected, these correlations were globally positive across participants (mean correlation: *r* = 0.62; Wilcoxon test: *T*(9) = 47, *p* = 0.048, BF_10_ = 3.00).

#### Metacognitive sensitivity

**Fig. 3:**
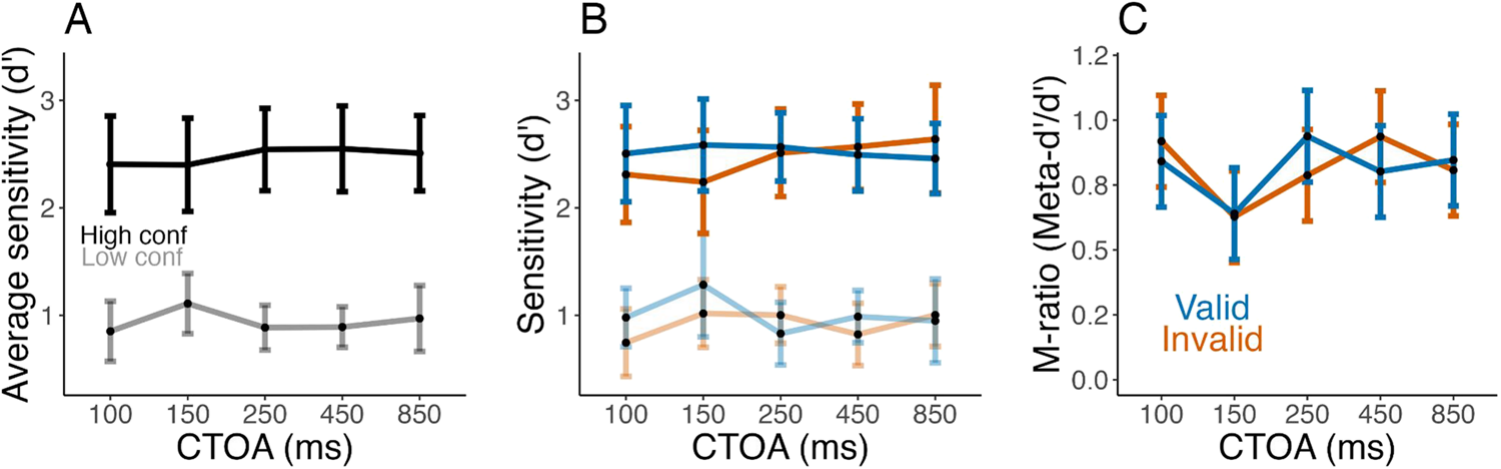
Cueing effects and metacognitive efficiency: (**A**) The average sensitivity for high (top trace, black) and low (bottom trace, grey) confidence trials, per cue-to-target onset asynchronies (CTOAs), a first measure of metacognition. Error bars present the mean and within-subject 95% confidence intervals (CIs). (**B**) Same as A but separately for valid (blue) and invalid (orange) conditions. (**C**) The metacognitive efficiency, “m-ratio” (the ratio meta-d’/d’) as a function of CTOAs and validity. Cueing does not significantly affect metacognitive efficiency, a result in line with the observed relationship between confidence and perceptual sensitivity. Error bars represent the mean and within-subject 95% CI

To check the presence of overall metacognitive insight, we compared participants’ perceptual sensitivity between high and low confidence trials. A repeated-measures ANOVA with sensitivity as the dependent variable and CTOA, validity, and confidence as independent variables indicated only a significant effect of confidence on sensitivity (*F*(1,9) = 76.59, *g* = 0.67, *p* < 0.001, BF_10_ = 1.67 × 10^42^), with no other main effects or interactions (all *p* > 0.09, BF_10_ < 0.02). In other words, when participants expressed higher confidence, their sensitivity was indeed higher, which indicates some metacognitive sensitivity (Fig. 3A and B).

#### Metacognition is stable across conditions

We quantified metacognitive efficiency (Fig. 3C) as the ratio of meta-d’ over d’ for each CTOA, cue validity condition and participant. Metacognitive efficiency appeared stable across conditions (Fig. 3C). An ANOVA on the m-ratio showed no significant effect of CTOA (*F*(1.7, 15.4) = 2.27, *g* = 0.06, *p* = 0.14, BF_10_ = 0.31) or validity (*F*(1,9) = 0.002, *g* < 0.001, *p* = 0.97, BF_10_ = 0.22), and no interaction (*F*(3.5, 31.3) = 0.9, *g* = 0.02, *p* = 0.6, BF_10_ = 0.14). Notably, Bayes factors provided evidence for an absence of effects, which is consistent with the interpretation that validity (or CTOA) affected both meta-d’ and d’ in a similar way, leading to a stable metacognitive efficiency. In other words, participants evaluated their performance adequately despite its fluctuation with cue validity and CTOA.

## Experiment 2

### Material and methods

#### Differences between Experiment 1 and Experiment 2

Experiment 1 demonstrated that exogenous cues affected both sensitivity and confidence. We devised a second, large-scale and pre-registered experiment to confirm our results. Because of COVID-19, Experiment 2 was conducted online. Five major changes were also introduced. First, instead of a binary discrimination task, participants now had to reproduce the orientation of the target. This continuous response was considered more engaging for online testing, since it avoided the need for a staircase procedure, and provided more information given that the outcome of interest becomes the magnitude of errors rather than their occurrence. Second, the number of CTOA conditions was reduced to just a short and a long CTOA, to keep the experiment short and facilitate online testing. Third, to ensure a covert orientation of attention, we included catch trials where a small target letter was displayed at fixation, which participants had to report. Fourth, to further encourage participants to keep their eyes on the center during the stimulus phase, we did not indicate the target location at stimulus onset, but rather at the end of the trial, an approach often found in the cueing literature as well (e.g., Pestilli & Carrasco, 2005). Finally, to mitigate any configural effect of the pre-cue on the perception of the target, the pre-cue was changed to a full circle surrounding the target.

#### Participants

One hundred and five adult volunteers were recruited via the Prolific online platform (age *M* ± *SD* = 29.3 ± 8.2 years, 76 females). The number of participants was determined following a separate preliminary study also conducted online (*N* = 23, see Online Supplementary Material (OSM)), in which the estimated effect size was *d* = 0.28. Power calculation (using G*power) indicated that at least 87 participants were needed to have an 85% power to detect the effect, we therefore defined *N* = 90 as a target. All participants provided electronic informed consent prior to the experiment. They were compensated 10 €/h for their time, plus a bonus of 2 € for the 50% best performing participants. An a priori < 80% correct-reporting exclusion criterion in catch trials was set to ensure covert orienting of attention. Following this rule, 15 out of the 105 participants were excluded, leading to 90 participants being kept for further analyses. The experiment consisted of one session (duration *M* ± *SD* = 52.3 ± 14.7 min). The experimental procedure was approved by the ethics review board of the Paris School of Economics (PSE). The study was pre-registered on AsPredicted.org (study #73304).

#### Stimuli

**Fig. 4:**
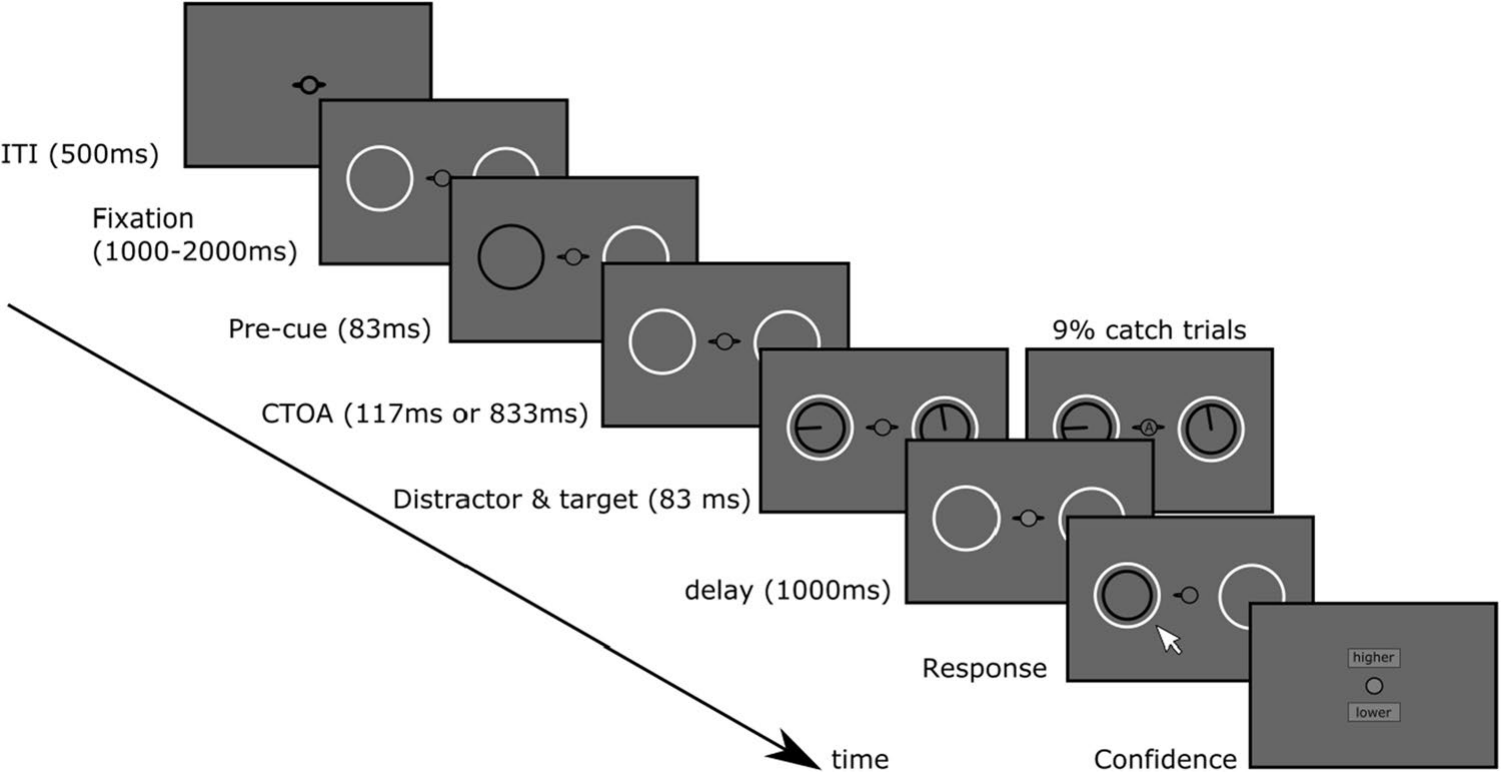
Experimental protocol for Experiment 2: On each trial, two white placeholders were presented on each side of a fixation point. After a random delay (sampled from a uniform distribution in the 1,000–2,000 ms range), one of the placeholders briefly turned black, to induce an exogenous orientation of attention. After a variable cue-to-target interval (CTOA), two clocks with random hand orientations were briefly presented within the placeholders. Following a 1-s delay, one of the two clocks was presented back, and the participant had to reproduce the orientation of the clock hand using the mouse cursor. Participants were informed that the pre-cue was random and not predictive of the target location. Once the response was given, the participant had to provide a confidence judgment. In 9% of the trials, a small target letter (‘A’ or ‘X’) was presented within the fixation point at clocks’ onset. In these trials, participants had to report both the target letter and the clock orientation. These responses were also followed by a confidence judgment

The experiment was displayed in full-screen mode. Exiting full-screen mode during the course of the experiment was automatically reversed in the following trial. Each trial started with a central fixation dot (0.6°) presented on a light gray background for 500 ms (inter-trial interval (ITI) period). Two black arrows (length: 0.6°) were systematically presented around the fixation dot, each pointing to two white circular placeholders located at a 3.5° eccentricity on each side of the fixation point, on the horizontal midline (2.1° diameter, see Fig. 4). The placeholders were displayed from the end of the ITI period to the end of the trial. A pre-cue, consisting of one of the placeholders briefly turning black, was presented for 83 ms randomly on the right or left side after a delay sampled from a uniform distribution (between 1,000 ms and 2,000 ms). While such a surrounding cue could potentially induce some masking (Luck et al., 1994; Yeshurun & Carrasco, 1999), therefore lowering the net observed exogenous effect, it minimizes possible interactions between the cue and the stimulus orientation (e.g., a dot flashed near the circle is not neutral with respect to orientation and may interact with the perceived orientation of the stimulus). Following one of two cue-to-target intervals (CTOAs, 117 ms or 833 ms), a target and a distractor were presented for 83 ms. It should be noted that our pre-registration erroneously mentioned 783 ms for our long CTOA in place of 833 ms in the present study. Target and distractor consisted of two clocks (black outline, 1.9° diameter) presented within the placeholders. For each clock, a single hand was presented at a random orientation, consisting of a line starting from the clock's center and connecting one point on the clock’s rim (length: 1.7°). One second after the clock’s offset, the response screen was presented, which featured a black circle at the target location, thereby indicating to the participant which stimulus was the target. The hand of the target clock was only displayed once the participant clicked on the clock in order to respond.

Figure 4 shows the experimental protocol for Experiment 2.

#### Procedure

Prior to starting the study, participants had to confirm their participation via a consent form. Then, to adapt the experiment to the characteristics of their screen, participants were presented with a standard visual adjustment procedure (Li et al., 2020). They had to rescale a rectangle using the mouse cursor to reproduce the size of a credit card held up on the screen. The estimated scaling factor was then used for the whole experiment, and allowed to present stimuli in standardized units. Following the procedure, participants were presented with instructions, followed by two demo trials in slow motion, involving both the reproduction task and the confidence judgment, and eight practice trials without the confidence judgment (to help them familiarize themselves with the average difficulty of the task).

Participants were instructed to fixate the center of the screen, to monitor the two placeholders, and to register the orientation of the two clocks’ hands displayed during the trial. They were informed that before the two clocks a brief black transient would appear randomly around the right or the left placeholder, that it was not predictive of cue location, and they were asked to simply ignore it. Participants had to report the phase of the target clock at the end of the trial using the mouse cursor. The mouse cursor was systematically hidden during the entire stimulus presentation phase. The target was randomly assigned as the right or left clock. The target was indicated by the clock’s outer-circle display and the fixation arrow during the response phase, that is, at the end of the trial (see Fig. 4). The display of the clock’s hand during the report period was initiated by the participant’s mouse click. To validate their response, participants were required to click on the fixation point, which turned blue following the participant's response. Participants were then asked to make a confidence judgment (“was the precision of your last response higher or lower than your average precision in this task?”), by clicking on one of two squares (2° × 4°) displayed at 2° eccentricity on the top and bottom of the fixation point, flanked by “Higher” and “Lower.” Once the confidence response was selected, the fixation dot turned blue, allowing participants to click on it. This approach was used to get the cursor and participants’ attention centered before the next trial would begin. Participants were not instructed to make speeded responses.

Participants were informed that some of the trials were catch trials (9% of the total). During these catch trials, a target letter (‘A’ or ‘X’, randomly selected, 0.2°) was presented for 83 ms within the fixation point, at the same time as the clocks, and participants were requested to report it after the phase of the target. Feedback on the letter discrimination task was given immediately after their response. Catch targets were randomly interleaved within the course of the experiment, and the CTOA and validity conditions for these trials were randomly assigned. At the end of each block, which lasted about 4 min, participants had the opportunity to take a 20-s break.

To minimize metacognitive bias (i.e., the tendency to always express “higher” or “lower” confidence on average), feedback was provided when participants' average confidence in the previous 20 trials was above 70% or below 30%. In these cases, participants were informed of the average confidence for the 20 previous trials and presented with a reminder of the instructions. The approach worked quite well, with a proportion of high confidence judgments not far from 50% overall (*M* ± *SD* = 58% ± 8%). Participants were informed that accuracy in clock reproduction will be considered for extra rewards calculation, and that failure to accurately report the target letter would disqualify them from being considered for extra rewards. Each participant completed 220 trials in total.

#### Analyses

Perceptual performance was measured using the average absolute orientation error. Hence, greater performance is synonymous with lower average absolute error, and vice versa. RTs were not considered of interest given that there was a 1-s delay between target offset and the prompt to respond. All analyses presented in the following section were pre-registered, unless otherwise stated. Catch trials were excluded from the analyses. As pre-registered, participants with a circular interquartile range (IQR) on clock reproduction error falling above or below 1.5 times the average IQR across participants were excluded, leaving 87 out of the 90 participants passing the catch trials criterion for further analysis. See the *Analyses* section of Experiment 1 for details on the statistical tests and conventions used.

### Results

#### Catch trials

Average performance on catch trials was high for both short (*M* ± *SD*, 92% ± 13%) and long (90% ± 16%) CTOA, with evidence for an absence of difference between CTOA conditions (*T*(86) = 1104, *r* = 0.07, *p* = 0.37, BF_10_ = 0.18, Wilcoxon test), suggesting that participants were not specifically making more eye movements in the long CTOA condition.

#### Confidence calibration and feedback

Only a limited proportion of blocks required feedback for confidence being too low (mean prop. ± SD = 1% ± 11%) or too high (11% ± 31%). This suggests that giving blockwise feedback can help participants to maintain balanced confidence reports. Nonetheless, there was a slight overconfidence in the estimation of the current trial: participants reported being more confident in the current trial (relative to the whole experiment) on 58% ± 8% of trials, that is, a little more than the normative value of 50%. Note that this tendency to be more confident for a single decision than for a whole set of decisions was also found for quiz questions (see, e.g., Gigerenzer et al., 1991).

#### Exogenous pre-cues affect performance and confidence

**Fig. 5:**
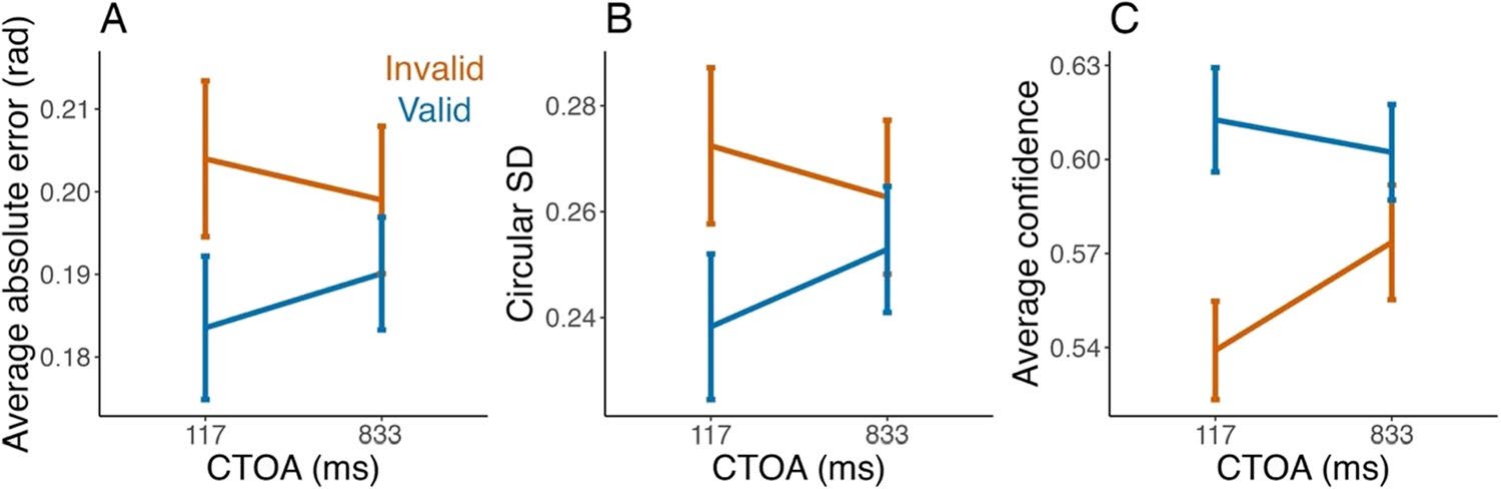
Cueing effects (Experiment 2): The (**A**) average absolute error (in radians), (**B**) circular standard deviation, and (**C**) average confidence, for the valid (blue) and invalid (orange) conditions, as a function of cue-to-target interval (CTOA). Average error and circular SD are significantly lower for the valid compared to invalid cues at the 117-ms CTOA, but not at the 833-ms CTOA. Average confidence is significantly higher in the valid compared to invalid conditions for both CTOAs. Error bars represent within-subjects 95% confidence intervals

Figure 5 shows the average performance and confidence per condition. As predicted, absolute error was lower for valid compared to invalid trials at the 117-ms CTOA (*T*(86) = 2,571, *r* = 0.30, *p* = 0.005, BF_10_ = 11.67, Wilcoxon test), but not at the 833-ms CTOA (*T*(86) = 2,114, *r* = 0.09, *p* = 0.40, BF_10_ = 0.20, Wilcoxon test), confirming a successful manipulation of exogenous attention. The positive evidence in favor of the null for the long CTOA also confirms the relative effectiveness of catch trials in maintaining fixation.

Following our pre-registration, we also considered circular standard deviation (SD) as a measure of performance (Fig. 5B), which produced similar results: circular SD was smaller for the valid compared to the invalid condition for the 117-ms CTOA (*T*(86) = 2,636, *r* = 0.33, *p* = 0.002, BF_10_ = 18.22, Wilcoxon test), but not for the 833-ms CTOA (*T*(86) = 2,040, *r* = 0.06, *p* = 0.60, BF_10_ = 0.16, Wilcoxon test).

For confidence (Fig. 5C), we found very strong evidence for a validity effect in the 117-ms CTOA (*t*(86) = 6.17, *d* = 0.70, *p* < 0.001, BF_10_ = 5.4 × 10^5^, Student’s t-test), but only negligible evidence in the 833-ms CTOA (*t*(86) = 2.28, *d* = 0.27, *p* = 0.02, BF_10_ = 1.35, Student’s t-test). We note that the average confidence was above, but not far, from 50% overall (*M* ± *SD* = 58% ± 8%).

#### Stable metacognition across conditions

**Fig. 6:**
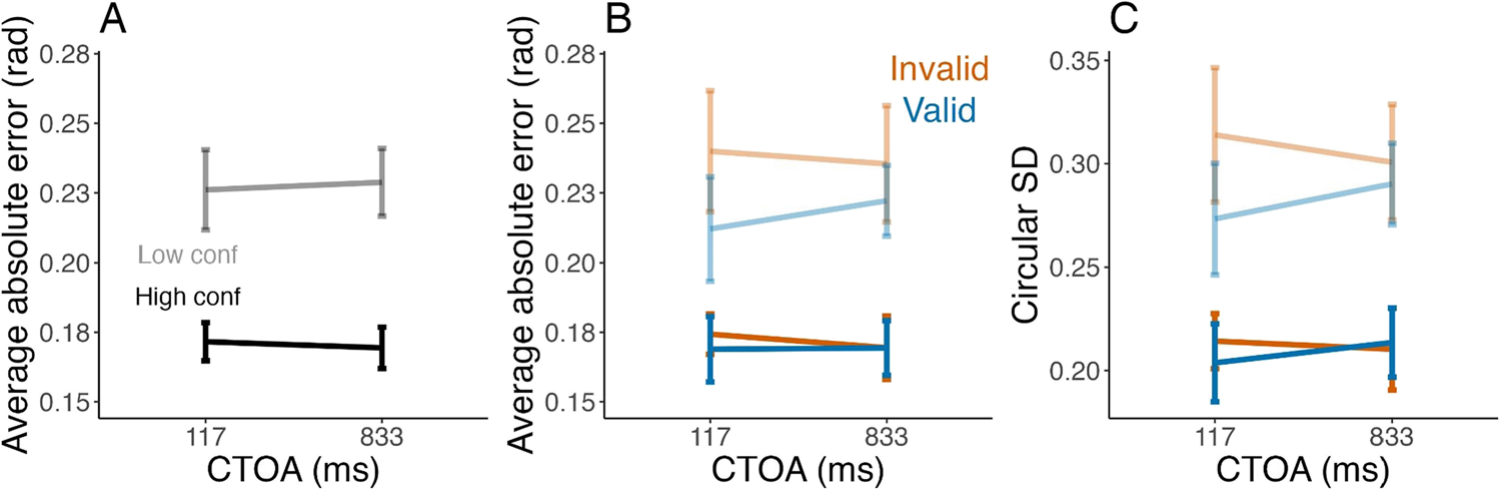
Metacognition of attention effect (Experiment 2): (**A**) The average absolute error (in radians) as a function of high (black) and low (grey) confidence as a function of cue-to-target interval (CTOA). Error bars represent the mean and within-subjects 95% confidence intervals (CIs). (**B**), (**C**) Similar plot, with high (bottom traces) and low (top traces) confidence subdivided into valid (blue) and invalid (orange) conditions for absolute error (**B**) and circular standard deviation (SD) (**C**). Error bars represent within-subjects 95% CIs

To measure metacognitive ability, we considered the difference in absolute error between high and low confidence trials (Fig. 6A, B). Based on Experiment 1, we expected in particular a main effect of confidence on error amplitude (i.e., confidence should be lower for larger errors), but no interaction between confidence and validity/CTOA. We found indeed a main effect of confidence (*F*(1,85) = 65.58, *g* = 0.11, *p* < 0.001, BF_10_ = 6.17 × 10^14^) and validity (*F*(1,85) = 4.60, *g* = 0.01, *p* = 0.035, BF_10_ = 0.36). There was no significant main effect of CTOA (*F*(1,85) = 0.01, *g* < 0.001, *p* = 0.942, BF_10_ = 0.08) and no significant interaction (confidence × validity : *F*(1,85) = 2.85, *g* = 0.003, *p* = 0.10, BF_10_ = 0.28; confidence × CTOA: *F*(1,85) = 0.82, *g* < 0.001, *p* = 0.37, BF_10_ = 0.14; validity × CTOA: *F*(1,85) = 0.97, *g* < 0.001, *p* = 0.33, BF_10_ = 0.16; confidence × validity × CTOA: *F*(1,85) = 0.28, *g* < 0.001, *p* = 0.60, BF_10_ = 0.28). For interactions involving confidence, Bayes factors indicated moderate evidence both for an absence of confidence × validity interaction, an absence of confidence × CTOA interaction as well as evidence for an absence of confidence × validity × CTOA interaction. Very similar results were found regarding the circular SD (Fig. 6C; see OSM for the ANOVA). In sum, these analyses show that the relation between confidence and performance was independent of CTOA and validity, metacognition remaining stable across cueing conditions.

## Discussion

In this study, we found that confidence judgments reflected the initial boost in sensitivity following involuntary shifts of attention, with greater confidence for valid than invalid pre-cues at brief cue-to-target intervals. The concurrent boost of sensitivity and confidence resulted in metacognitive ability remaining largely stable under fluctuations of exogenous attention.

In theory, the effect of exogenous attention on confidence could result from either a shift in confidence bias (i.e., the average confidence in a given condition) or sensitivity, or a mixture of both (Mamassian, 2016). In Experiment 2, we used a continuous measure of performance, and observed smaller errors for high confidence trials, both within and across valid/invalid trials. This result confirms that confidence has some access to the fluctuations in sensory uncertainty shaping the estimation response, and does not merely reflect a heuristic favoring valid cues. Participants could have relied on both sensory evidence and motor response when judging the precision of their estimate, yet the motor component should have affected all trials similarly, leaving most of the observed effect to sensory modulation. Of note, while in Experiment 1 the target location was immediately known, it was indicated at the end of the trial in Experiment 2: therefore, each experiment involved a distinct type of memory retrieval, making our conclusions generalizable to different settings.

A previous paper that investigated whether exogenous pre-cueing could influence confidence found no effect (Kurtz et al., 2017). However, the pre-cues used in that paper were always valid (compared to a no-cue condition) and only one CTOA (90–110 ms) was used, thus making it difficult to rule out non-exogenous and expectation effects. In the present study, using non-predictive valid/invalid pre-cues and several CTOAs, we could control for these aspects. We found that valid pre-cue increased both sensitivity and confidence at short CTOAs compared to invalid pre-cues. These effects were observed despite participants being clearly informed that cue location was randomly drawn, and that there was no reason to expect that the target would appear at the same location. Participants seem to have understood these instructions, since for longer cue-to-target intervals sensitivity was similar between the valid and invalid locations (suggesting that participants did not reallocate their attention voluntarily where the pre-cue appeared).

In addition, we also used standard measures to assess metacognition, and we did not find evidence for a detrimental effect of cueing on metacognition, a result coherent with the notion that metacognitive ability remains stable following the combined manipulation of endogenous and exogenous attention (Landry et al., 2021). In their study, Landry et al., concurrently used exogenous/endogenous cues and found confidence to reflect changes in sensitivity. Yet, the existence of two types of cues (exogenous and endogenous) in their manipulation could have had a specific effect on metacognitive processing above and beyond the independent effects of each type of attention. Using a purely exogenous paradigm, our results therefore confirm a robust metacognitive tracking of transient attention, in the absence of any voluntary component.

The observed early boost in sensitivity and confidence might come from a facilitation at the valid location, a suppression at the invalid location, or a mixture of both (Carrasco, 2011). One approach to tackle the selectivity of the cueing process might be to use trials with neutral cues (e.g., where both locations are cued simultaneously). Whether confidence is equally sensitive to the suppression and facilitation induced by pre-cueing is a question for further work to address.

These results suggest that the gain in visual processing induced with spatial uninformative transients can be introspected. It has been suggested that confidence accurately tracks the effect of voluntary, endogenous attention (Denison et al., 2018; Kurtz et al., 2017; Zizlsperger et al., 2012; but see Wilimzig et al., 2008). Yet, we recently found the orienting of endogenous attention to induce a transitory metacognitive impairment at deployment onset, while the orienting of exogenous attention did not produce such an impairment (Recht et al., 2021). This result highlights the need for a thorough investigation on how the attentional system interacts with metacognition at different levels of cognitive control. The present results confirm a robust metacognition both at the peak and waning of exogenous attention: during involuntary shifts of attention, and despite most of the changes in evidence remaining beyond top-down control, metacognition keeps an accurate representation of the underlying sensory fluctuations. Of note, we are not making the claim that metacognition has an online, direct access to the changes in perception that occur at the exact time of appearance of the stimulus or the attentional cue. Confidence reports being collected well after the stimulus is gone, we can only argue that metacognition has access, at least, to the outcome of sensory and attentional processes driving perceptual decisions.

Visual confidence has been proposed to play a role in numerous decisional processes, including adaptive learning (Guggenmos et al., 2016; Hainguerlot, Vergnaud, & De Gardelle, 2018; Zylberberg, Wolpert, & Shadlen, 2018), task prioritization (Aguilar-Lleyda, Lemarchand, & de Gardelle, 2020) and evidence accumulation (Balsdon, Wyart, & Mamassian, 2020; Desender, Boldt, & Yeung, 2018). Confidence has also been regarded as a form of common, supramodal currency for the perceptual system (de Gardelle, Le Corre, & Mamassian, 2016; de Gardelle & Mamassian, 2014). Attention having a substantial role in shaping perception and decision, an accurate reflection of attention in metacognition is critical. The present work suggests that metacognition efficiently tracks the effects of a reflexive attentional mechanism known to evade voluntary control, thus illustrating a striking ability of high-level cognition to capture fleeting, low-level sensory modulations.

## Supplementary information

ESM 1 (DOCX 626 kb)

## Data Availability

The data for Experiment 1 are freely available as part of the Confidence database (Rahnev et al., 2020), via Open Science Framework: https://www.osf.io/s46pr/. The data for Experiment 2 are also available via Open Science Framework: https://www.osf.io/gczq8/. The pre-registration for Experiment 2 is available on AsPredicted.org (study #73304): https://www.aspredicted.org/qm43r.pdf.

## References

[CR1] Aguilar-Lleyda D, Lemarchand M, de Gardelle V (2020). Confidence as a Priority Signal. Psychological Science.

[CR2] Baguley, T. (2012). Calculating and graphing within-subject confidence intervals for ANOVA. *Behavior Research Methods*. 10.3758/s13428-011-0123-7 10.3758/s13428-011-0123-7 21858605

[CR3] Balsdon T, Wyart V, Mamassian P (2020). Confidence controls perceptual evidence accumulation. Nature Communications.

[CR4] Carrasco, M. (2011). Visual attention: The past 25 years. *Vision Research*. 10.1016/j.visres.2011.04.012 10.1016/j.visres.2011.04.012 PMC3390154 21549742

[CR5] Cousineau, D. (2005). Confidence intervals in within-subject designs: A simpler solution to Loftus and Masson’s method. *Tutorials in Quantitative Methods for Psychology*. 10.20982/tqmp.01.1.p042

[CR6] de Gardelle V, Mamassian P (2014). Does Confidence Use a Common Currency Across Two Visual Tasks?. Psychological Science.

[CR7] de Gardelle V, Le Corre F, Mamassian P (2016). Confidence as a Common Currency between Vision and Audition. PloS One.

[CR8] Denison, R. N., Adler, W. T., Carrasco, M., & Ma, W. J. (2018). Humans incorporate attention-dependent uncertainty into perceptual decisions and confidence. *Proceedings of the National Academy of Sciences*, 201717720. 10.1073/pnas.1717720115 10.1073/pnas.1717720115 PMC6205425 30297430

[CR9] Desender, K., Boldt, A., & Yeung, N. (2018). Subjective Confidence Predicts Information Seeking in Decision Making. *Psychological Science*. 10.1177/0956797617744771 10.1177/0956797617744771 29608411

[CR10] Fernández A, Li HH, Carrasco M (2019). How exogenous spatial attention affects visual representation. Journal of Vision.

[CR11] Gigerenzer G, Hoffrage U, Kleinbölting H (1991). Probabilistic mental models: a Brunswikian theory of confidence. Psychological Review.

[CR12] Gilbert CD, Li W (2013). Top-down influences on visual processing. Nature Reviews Neuroscience.

[CR13] Green DG, Swets JA (1966). Signal detection theory and psychophysics.

[CR14] Guggenmos, M., Wilbertz, G., Hebart, M. N., & Sterzer, P. (2016). Mesolimbic confidence signals guide perceptual learning in the absence of external feedback. *ELife*. 10.7554/eLife.13388 10.7554/eLife.13388 PMC4821804 27021283

[CR15] Hainguerlot, M., Vergnaud, J. C., & De Gardelle, V. (2018). Metacognitive ability predicts learning cue-stimulus associations in the absence of external feedback. *Scientific Reports*. 10.1038/s41598-018-23936-9 10.1038/s41598-018-23936-9 PMC5884814 29618809

[CR16] Jonides J, Irwin DE (1981). Capturing attention. Cognition.

[CR17] Kesten H (1958). Accelerated Stochastic Approximation. The Annals of Mathematical Statistics.

[CR18] Kiani R, Shadlen MN (2009). Representation of confidence associated with a decision by neurons in the parietal cortex. Science.

[CR19] Kurtz, P., Shapcott, K. A., Kaiser, J., Schmiedt, J. T., & Schmid, M. C. (2017). The Influence of Endogenous and Exogenous Spatial Attention on Decision Confidence. *Scientific Reports, 7*(1). 10.1038/s41598-017-06715-w 10.1038/s41598-017-06715-w PMC5527098 28743958

[CR20] Landry M, Da Silva Castanheira J, Sackur J, Raz A (2021). Investigating how the modularity of visuospatial attention shapes conscious perception using type I and type II signal detection theory. Journal of Experimental Psychology. Human Perception and Performance.

[CR21] Li Q, Joo SJ, Yeatman JD, Reinecke K (2020). Controlling for participants’ viewing distance in large-scale psychophysical online experiments using a virtual chinrest. Scientific reports.

[CR22] Ling, S., & Carrasco, M. (2006). Sustained and transient covert attention enhance the signal via different contrast response functions. *Vision Research*. 10.1016/j.visres.2005.05.008 10.1016/j.visres.2005.05.008 PMC1557421 16005931

[CR23] Liu T, Pestilli F, Carrasco M (2005). Transient Attention Enhances Perceptual Performance and fMRI Response in Human Visual Cortex. Neuron.

[CR24] Luck SJ, Hillyard SA, Mouloua M, Woldorff MG, Clark VP, Hawkins HL (1994). Effects of spatial cuing on luminance detectability: psychophysical and electrophysiological evidence for early selection. Journal of experimental psychology: human perception and performance.

[CR25] Macmillan NA, Creelman CD (2005). *Detection Theory: A User’s Guide*. *Detection theory: A user’s guide*.

[CR26] Mamassian P (2016). Visual Confidence. Annual Review of Vision Science.

[CR27] Maniscalco B, Lau H (2012). A signal detection theoretic approach for estimating metacognitive sensitivity from confidence ratings. Consciousness and Cognition.

[CR28] Morey, R. D. (2008). Confidence Intervals from Normalized Data: A correction to Cousineau (2005). *Tutorials in Quantitative Methods for Psychology.*10.20982/tqmp.04.2.p061

[CR29] Morey, R. D., & Rouder, J. N. (2018). BayesFactor: Computation of Bayes factors for Common Designs. Retrieved from https://cran.r-project.org/package=BayesFactor

[CR30] Nakayama K, Mackeben M (1989). Sustained and transient components of focal visual attention. Vision Research.

[CR31] Nobre A, Kastner S (2014). The oxford handbook of attention.

[CR32] Peirce JW (2007). PsychoPy-Psychophysics software in Python. Journal of Neuroscience Methods.

[CR33] Pestilli F, Carrasco M (2005). Attention enhances contrast sensitivity at cued and impairs it at uncued locations. Vision Research.

[CR34] Posner, M. I. (1980). Orienting of attention. *Quarterly Journal of Experimental Psychology*. 10.1080/00335558008248231 10.1080/00335558008248231 7367577

[CR35] R Core Team. (2013). R: A Language and Environment for Statistical Computing. Retrieved from http://www.r-project.org/

[CR36] Rahnev, D., & Fleming, S. M. (2019). How experimental procedures influence estimates of metacognitive ability. *Neuroscience of Consciousness*. 10.1093/nc/niz009 10.1093/nc/niz009 PMC6556214 31198586

[CR37] Rahnev, D., Desender, K., Lee, A. L. F., Adler, W. T., Aguilar-Lleyda, D., Akdoğan, B., … Zylberberg, A. (2020). The confidence database. *Nature Human Behaviour*, *4*(3), 317-325. 10.1038/s41562-019-0813-1 10.1038/s41562-019-0813-1 PMC7565481 32015487

[CR38] Recht, S., Mamassian, P., & de Gardelle, V. (2019). Temporal attention causes systematic biases in visual confidence. *Scientific Reports, 9*(1), 11622. 10.1038/s41598-019-48063-x PMC6690997 31406265

[CR39] Recht, S., de Gardelle, V., & Mamassian, P. (2021). Metacognitive blindness in temporal selection during the deployment of spatial attention. *Cognition, 216*, 104864. 10.1016/j.cognition.2021.104864 34339907

[CR40] Remington RW, Johnston JC, Yantis S (1992). Involuntary attentional capture by abrupt onsets. Perception & Psychophysics.

[CR41] Solomon, J. A., & Morgan, M. J. (2018). Precues’ elevation of sensitivity is not only preattentive, but largely monocular. *Attention, Perception, and Psychophysics*. 10.3758/s13414-018-1564-1 10.3758/s13414-018-1564-1 PMC6153965 29987533

[CR42] White AL, Lunau R, Carrasco M (2014). The attentional effects of single cues and color singletons on visual sensitivity. Journal of Experimental Psychology: Human Perception and Performance.

[CR43] Wilimzig C, Tsuchiya N, Fahle M, Einhäuser W, Koch C (2008). Spatial attention increases performance but not subjective confidence in a discrimination task. Journal of Vision.

[CR44] Yeshurun Y, Carrasco M (1999). Spatial attention improves performance in spatial resolution tasks. Vision research.

[CR45] Zizlsperger, L., Sauvigny, T., & Haarmeier, T. (2012). Selective attention increases choice certainty in human decision making. *PLoS ONE*. 10.1371/journal.pone.0041136 10.1371/journal.pone.0041136 PMC3397971 22815942

[CR46] Zylberberg, A., Wolpert, D. M., & Shadlen, M. N. (2018). Counterfactual reasoning underlies the learning of priors in decision making. *Neuron*. 10.1016/j.neuron.2018.07.035 10.1016/j.neuron.2018.07.035 PMC6127036 30122376

