# Supplementary material for: Metacognition tracks sensitivity following involuntary shifts of visual attention
Source: Psychon Bull Rev. 2022 Nov 16;30(3):1136–47. doi: 10.3758/s13423-022-02212-y (PMC9668230; doi:10.3758/s13423-022-02212-y)

Metacognition tracks sensitivity following involuntary shifts of visual attention

Supplementary Material

## Pilot study for Experiment 2

In Experiment 1, we used a classic psychophysical paradigm to probe exogenous attention. The paradigm required a staircase procedure to adjust target features per observer. Such an approach is less practical in an online setting, and we therefore devised a distinct paradigm involving a reproduction task. The advantage of a reproduction task is twofold: first, it provides a better signal-to-noise ratio per trial, since the response is continuous rather than binary; second, it relaxes the need for tailored difficulty levels, as it is not the probability of an error, but its magnitude, that is of interest. In other words, the risk of ceiling effects is vastly diminished.

Because this approach is less prevalent in the literature on exogenous attention, we decided to conduct a preliminary study to estimate the effect size. The preliminary study was virtually equivalent to Exp. 2 described in the main paper, but involved a smaller number of participants (N = 23, from a distinct pool of participants), and was not pre-registered.

### Participants

23 adult volunteers were recruited via the Prolific online platform (age M ± SD = 32 ± 10-year-old, 13 females). All participants provided informed electronic consent prior to the experiment. They were compensated 10€/hour for their time, plus a bonus of 2 € for the 50% best performing participants. One participant had a technical difficulty and was excluded due to missing data. An a priori <80% exclusion criterion in catch trials was set to ensure covert orienting of attention. Following this rule, 2 out of the 22 participants were excluded, leading to 20 participants being kept for further analyses. The experiment consisted of one session (duration M ± SD = 65 ± 15 min). The experimental procedure was approved by the ethics review board of the Paris School of Economics (PSE).

### Results

Outliers with a circular IQR on clock reproduction error above/below 1.5 times the group IQR were excluded, leaving 18 participants for further analysis.

Figure S1 shows the average performance and confidence per condition. We did not find the average absolute error to be lower for the valid, compared to invalid condition neither at the 117ms CTOA (*t*(17) = 1.62, *d* = 0.42, *p* = 0.12, BF_10_ = 0.74, Student t-test), nor at the 833ms CTOA (*t*(17) = 1.35, *p* = 0.19, *d* = 0.28, BF_10_ = 0.53). We therefore preregistered the lower of the two estimated effect sizes (*d* = 0.28) as a target for our power analysis in Exp 2.

For confidence, we found strong evidence for a validity effect in the 117ms CTOA (*t*(17) = 3.90, *d* = 1.04, *p* = 0.001, BF_10_ = 33.3, Student t-test) but evidence favoring the null hypothesis in the 833ms CTOA (*t*(17) = 0.03, *d* = 0.01, *p* = 0.17, BF_10_ = 0.24, Student t-test).


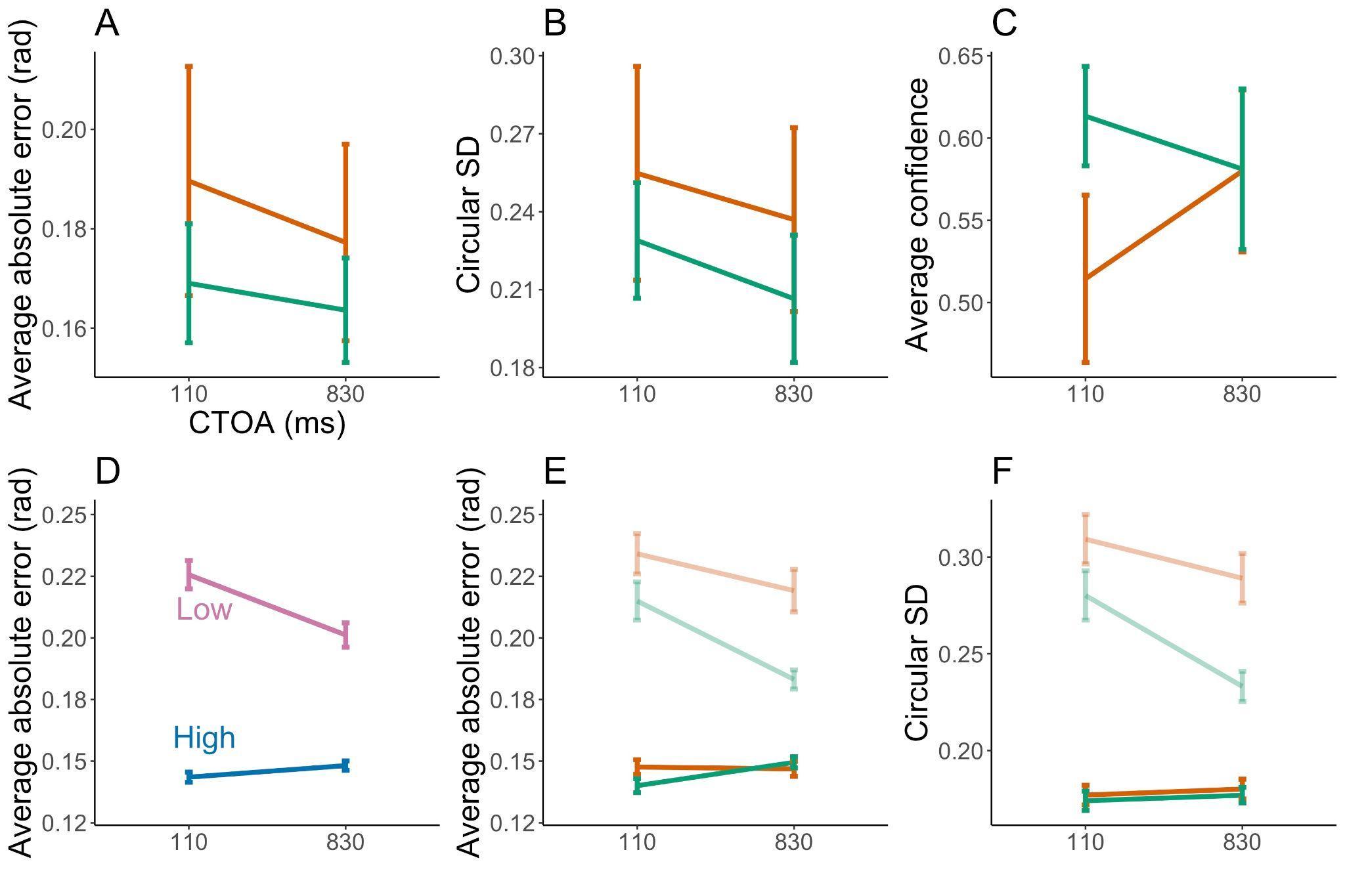


Figure S1: **Results for the pilot study of Experiment 2**. The (A) average absolute error (in radians), (B) circular standard deviation, and (C) average confidence, for the valid (green) and invalid (red) conditions, as a function of CTOA. Error bars represent within-subjects, 95% CI.

## Confidence & circular SD in Experiment 2

The analysis of metacognitive ability using the circular SD in place of the absolute error virtually mirrored the results found using the latter. We found a main effect of confidence (*F*(1,85) = 68.26, *g* = 0.11, *p* < 0.001, BF_10_ = 7.54 x 10^14^) and validity (*F*(1,85) = 4.08, *g* = 0.004, *p* = 0.047, BF_10_ = 0.25). There was no significant main effect of CTOA (*F*(1,85) = 0.01, *g* < 0.001, *p* = 0.921, BF_10_ = 0.09) and no significant interaction (confidence x validity : *F*(1,85) = 2.45, *g* = 0.002, *p* = 0.12, BF_10_ = 0.21; confidence x CTOA: *F*(1,85) = 0.02, *g* < 0.001, *p* = 0.90, BF_10_ = 0.12; validity x CTOA: *F*(1,85) = 3.29, *g* = 0.002, *p* = 0.073, BF_10_ = 0.21; confidence x validity x CTOA: *F*(1,85) = 0.64, *g* < 0.001, *p* = 0.43, BF_10_ = 0.20). For interactions involving confidence, Bayes factors indicated moderate evidence both for an absence of confidence x validity interaction, an absence of confidence x CTOA interaction as well as evidence for an absence of confidence x validity x CTOA interaction.

## Trial-by-trial analysis for Experiment 2

To confirm that our conclusions generalize to trial-by-trial fluctuation in error and confidence, we also used an alternative, mixed-effects logistic regression approach, and compared different models. Confidence judgment (this time, at the trial level, that is, ‘high’ or ‘low’), was predicted by the absolute error plus the different factors as fixed effects. Participants were considered as random intercepts in the model. Only two models were outperforming the null model in predicting confidence: a model with the absolute error as main effect (χ^2^(1) = 288.55, *p* < 0.001, ΔBIC = -278), and a model with absolute error and validity as main effects (χ^2^(2) = 329.89, *p* < 0.001, ΔBIC = -310), the latter also outperforming the former (χ2(1) = 41.32, *p* < 0.001, ΔBIC = -32). These results suggest that participants were able to introspect the magnitude of their error at the trial level and to use this information during their confidence judgment.

## Individual figures for the cueing effect in Exp 1 and 2

To have a better view on the individual trends, the cueing effect (valid-invalid) for sensitivity and confidence (valid-invalid) in Exp 1 and error (invalid-valid) and confidence (valid-invalid) in Exp 2 were estimated per CTOA.

### Experiment 1

The x-axis corresponds to the cueing effect on confidence, calculated as the confidence in valid minus invalid trials. The y-axis corresponds to the cueing effect on performance, calculated as d’ in valid minus invalid trials. Each individual point represents a CTOA, and the blue line a regression (the shaded band represents the 95 % CI).


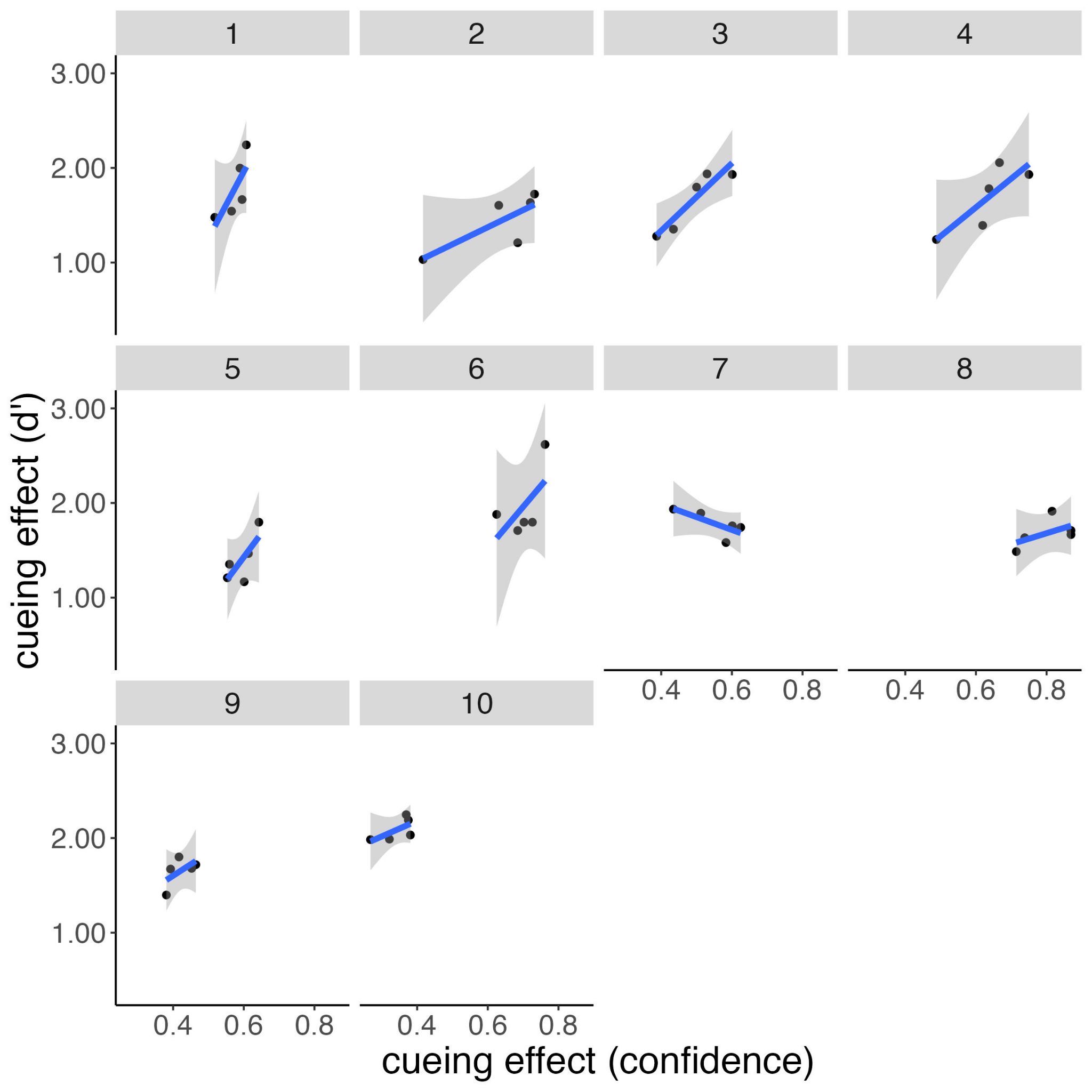


### Experiment 2

The x-axis corresponds to the cueing effect on confidence, calculated as the confidence in valid minus invalid trials. The y-axis corresponds to the cueing effect on performance, calculated as error in invalid minus valid trials. Each panel shows the data of a single participant, the two dots correspond to the cueing effects in the two CTOAs.


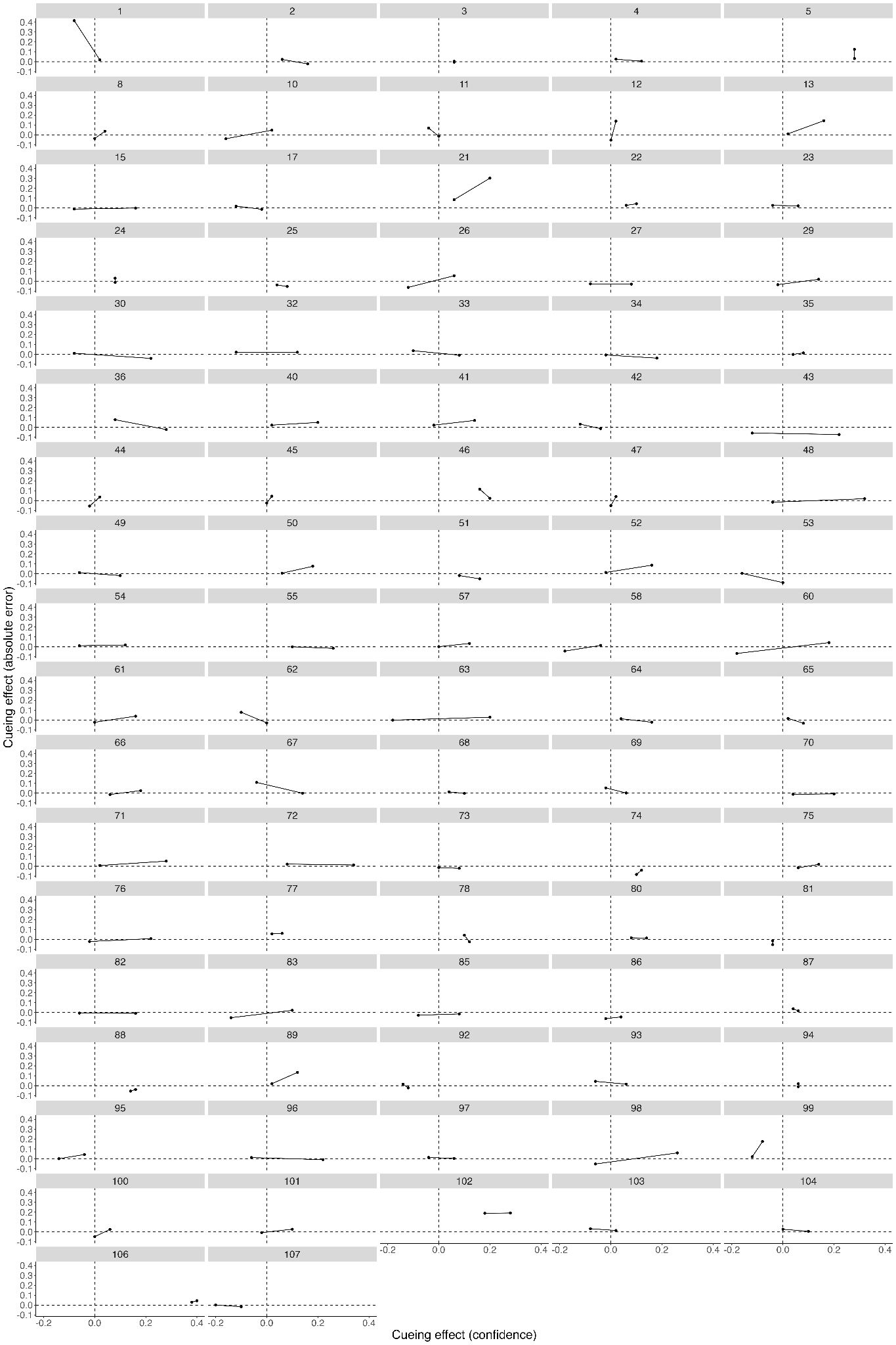

Supplement: Supplementary file 1 — (DOCX 626 kb) [file 13423_2022_2212_MOESM1_ESM.docx]
